# Antibacterial and Anticancer Activities of Pleurocidin-Amide, a Potent Marine Antimicrobial Peptide Derived from Winter Flounder, *Pleuronectes americanus*

**DOI:** 10.3390/md20080519

**Published:** 2022-08-14

**Authors:** Hui-Chen Hsu, Ming-Hsin Chen, Ming-Lung Yeh, Wei-Jung Chen

**Affiliations:** 1 Department of Biotechnology and Animal Science, National Ilan University, Yilan 26047, Taiwan; 2 Department of Laboratory Medicine, Yuanshan Branch, Veterans General Hospital, Yilan 264, Taiwan

**Keywords:** Pleurocidin (Ple), antimicrobial peptide (AMP), antibacterial, anticancer, multidrug resistance (MDR), non-small cell lung adenocarcinoma

## Abstract

The extensive use of conventional antibiotics has led to the growing emergence of many resistant strains of pathogenic bacteria. Evidence suggests that cationic antimicrobial peptides (AMPs) have the greatest potential to serve as traditional antibiotic substitutes. Recent studies have also reported that certain AMPs have selective toxicity toward various types of cancer cells. The electrostatic attraction between the negatively charged membrane components and AMPs is believed to play a crucial role in the disruption of bacterial and cancer cell membranes. In the current study, we used a potent AMP called Pleurocidin (Ple) derived from winter flounder *Pleuronectes americanus* and its C-terminal-amidated derivative Pleurocidin-amide (Ple-a), and evaluated their antibacterial and anticancer activities. Our results indicated that both Ple and Ple-a exhibited significant antibacterial activity against a broad spectrum of Gram-positive and Gram-negative bacteria, especially marine pathogens, with MIC values ranging from 0.25 to 32 μg/mL. These peptides are also potent against several multidrug-resistant (MDR) bacterial strains, with MIC values ranging from 2 to 256 μg/mL. When used in combination with certain antibiotics, they exhibited a synergistic effect against MDR *E. coli*. Ple and Ple-a also showed notable cytotoxicity toward various cancer cell lines, with IC_50_ values ranging from 11 to 340 μM, while normal mouse fibroblast 3T3 cells were less susceptible to these peptides. Ple-a was then selected to study its anticancer mechanism toward A549 human lung adenocarcinoma cells. Western blot analysis and confocal microscopy showed that Ple-a could inhibit autophagy of A549 cells, and induce apoptosis 48 h after treatment. Our findings provided support for the future application of Ple-a as potential therapeutic agent for bacterial infections and cancer treatment.

## 1. Introduction

Extensive use of conventional antibiotics has led to the growing emergence of many resistant bacterial pathogens [1,2]. Therefore, the development of novel antibacterial agents that could conquer the resistance problem has become crucial. Evidence suggests that antimicrobial peptides (AMPs) have the greatest potential to serve as alternatives to classical antibiotics [3,4,5]. AMPs are small peptides predominantly composed of amphipathic amino acid sequences with a positive net charge, and are important components of the innate immune system and host defense mechanism of most living organisms [6,7]. One of the ways that AMPs are classified is based on the secondary structures they adopt upon contact with biological membranes. The largest group of AMPs reported in peptide databases consists of peptides that fold into an amphipathic α-helical conformation when interacting with their targets [8,9] Most of them exert their activity by disrupting bacterial membrane or by interfering with intracellular processes. Structurally random in solution, these AMPs fold into an amphipathic helix upon binding and insertion into the target membrane, leading to breakdown of the transmembrane potential and ion gradients, thus causing leakage of cell contents and finally resulting in cell death [10].

The skin and skin mucus of several fish species have been shown to contain AMPs. Pleurocidin (Ple) is a well-known α-helical cationic AMP isolated from the skin mucous secretion of winter flounder, *Pleuronectes americanus* [11,12,13]. Ple consists of 25 amino acid residues with a net positive charge at physiological pH. Previous structural studies using circular dichroism (CD) spectroscopy have shown that in the presence of trifluoroethanol (TFE), sodium dodecyl sulfate (SDS), and various types of membrane models, Ple adopts an α-helical structure with hydrophilic and hydrophobic residues on opposing sides of the helical structure [14,15,16]. Ple has a broad spectrum of antibacterial activity against Gram-positive and Gram-negative bacteria, while it has very low hemolytic activity against red blood cells [17,18,19,20]. Ple could also affect various intracellular processes, indicating the multiple activity mechanisms of the peptide [21,22]. Former studies have also demonstrated that the peptide has strong interactions with anionic lipids (characteristics of bacterial membrane), while it weakly interacts with zwitterionic lipids (mimicking eukaryotic cell membrane) [17]. Cho et al. (2012) prepared several partial deletion analogues of Ple and the results showed that the N- and C-terminal regions are crucial for its capability to interact with and penetrate the cell membrane [23]. Choi and Lee (2013) also provided evidence that the N- and C-terminal truncation variants of Ple have lost their ability to induce apoptosis in fungus *Candida albicans*, indicating that the N- and C-terminal region of Ple plays an important role in its antifungal activity [24].

Since Ple has selective lipid membrane-perturbation activities [25,26], it is a suitable candidate for use in the treatment of bacterial infections and cancer therapy. Previous studies have also indicated that the peptide shows cytotoxicity to breast and myeloma carcinoma cells [27,28] and inhibits the growth of tumor xenografts [28,29]. However, whether Ple is effective against multidrug-resistant (MDR) bacteria remains unknown. In this study, we intended to investigate whether Ple and its derivative are able to exhibit antibacterial effect against MDR bacterial strains, and further delineate its anticancer efficacy and mechanism against the non-small cell lung adenocarcinoma A549 cell line.

## 2. Results

### 2.1. Characteristics of Pleurocidin (Ple) and Its C-terminal Amidation Derivative Ple-a

**Figure 1:**
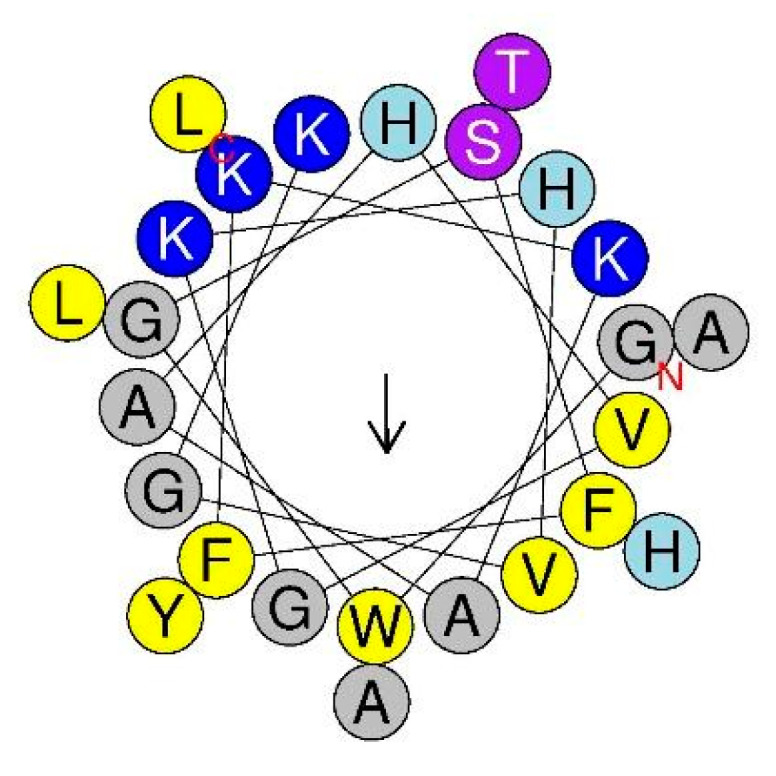
Helical-wheel diagrams of Ple using HeliQuest (http://heliquest.ipmc.cnrs.fr/ (accessed on 23 April 2021)). Positively charged residues (K and H) are shown in dark and light blue. Hydrophilic residues (S and T) are in magenta. Hydrophobic residues (W, Y, F, L, V) are shown in yellow, and G and A are shown in gray. The arrow indicates the orientation of the hydrophobic moment. C and N denote the C- and N-terminal residues.

In our previous reports, we designed and synthesized a series of novel cationic antimicrobial peptides (AMPs) [30,31]. In these studies, Pleurocidin (Ple) from winter flounder was applied as positive control. Ple is a typical cationic amphipathic AMP with an α-helix structure, as shown by its helical wheel diagram (Figure 1). We also investigated the sequence characteristics of Ple (Table 1) and performed sequence modification of this natural AMP (data not shown). A series of N-terminal and C-terminal truncation derivatives were obtained and their antibacterial activity assayed. Surprisingly, only full-length Ple possessed antibacterial activity against several typical bacterial strains, while all truncated derivatives lost their antibacterial activity (data not shown). According to former studies [32], C-terminal amidation may provide more positive net charge to AMPs, thus increasing their binding affinity to negatively charged bacterial membranes, resulting in enhanced antimicrobial activity. In the current study, Ple and its C-terminal amidated derivative Ple-a were selected and their antibacterial and anticancer activity were further investigated.

### 2.2. Antibacterial Activity of Ple and Ple-a against Gram-Positive and Gram-Negative Bacteria

We determined the antibacterial activity of Ple and Ple-a against a broad spectrum of Gram-positive and Gram-negative microbes by measuring the minimal inhibitory concentrations (MICs) using a standard serial dilution method [30]. As shown in Table 2, Ple and Ple-a exhibited higher antibacterial activity against Gram-negative bacteria (MIC values of 0.5~64 μg/mL) as compared with those against Gram-positive strains (MIC values of 4~128 μg/mL). Meanwhile, Ple-a displayed better antibacterial activity (MIC values of 0.5~32 μg/mL) than that of Ple (MIC values of 1~128 μg/mL), indicating that C-terminal amidation results in a more positive net charge of the peptide and contributes to its antibacterial activity. Furthermore, seven important marine pathogens were also included in the study, and Ple and Ple-a showed potent antibacterial activity against these bacteria with MIC values of Ple (1~128 μg/mL) and Ple-a (0.5~32 μg/mL). Both peptides were more active against Gram-negative marine pathogens including *Vibrio alginolyticus, V. anguillarum serotype O1, V. parahaemolyticus*, *V. harveyi*, and *Photobacterium damselae subsp. piscicida* (MIC values of 1~8 μg/mL)*,* while they were less active against Gram-positive strains such as *Streptococcus iniae* and *Lactococcus garvieae* (MIC values of 32~128 μg/mL).

In order to verify whether Ple and Ple-a are also effective against multidrug-resistant (MDR) bacterial pathogens, several strains were collected from National Yang-Ming Chiao-Tung University Hospital located in Yilan City, including *Enterococcus faecium*-4R, *Escherichia coli*-7R, *Pseudomonas aeruginosa*-5R, *Klebsiella pneumoniae*-7R, *Klebsiella pneumoniae*-10R, and *Acinetobacter baumannii*-8R. Another strain, *Salmonella enterica* serovar Choleraesuis-13R, was obtained from the Animal Health Research Institute, Council of Agriculture, Executive Yuan, Taiwan. Both peptides showed potent antibacterial activity against these MDR bacterial strains, with MIC values of 8~256 μg/mL (Ple) and 2~32 μg/mL (Ple-a).

### 2.3. Synergistic Eeffect of Ple-a Used in Combination with Antibiotics against MDR E. coli

Among all MDR bacterial strains tested, Ple-a showed the most potent antibacterial activity against *Escherichia coli*-7R with an MIC value of 2 μg/mL (Table 2). The peptide was then used in combination with several conventional antibiotics including penicillin (Ampicillin), aminoglycosides (Amikacin and Gentamycin), carbapenems (Imipenem and Meropenem), cephalosporins (Ceftazidime and Cefotaxime), and fluoroquinolone (Levofloxacin) and their MIC values and fractional inhibition concentrations indexes (FICI) against MDR *E. coli*-7R were measured (Table 3) using the checkerboard titration method according to a previous report [34]. It was found that when Ple-a and antibiotics were used at a ratio of 1:1, Ampicillin, Cefotaxime, and Gentamycin showed synergistic effects. When Ple-a and antibiotics were used at a ratio of 2:1, Ampicillin, Imipenem, Ceftazidime, Cefotaxime, and Gentamycin showed synergistic effects. However, when Ple-a and antibiotics were used at a ratio of 1:2, only Cefotaxime showed a synergistic effect (Table 3). It is evident that Ple-a showed synergistic effects with a broad spectrum of antibiotics in a dose-dependent manner.

### 2.4. Selective Cytotoxicity of Ple and Ple-a against A549 and NIH-3T3 Cell Lines

In order to understand the cytotoxic effect of Ple and Ple-a against typical cancer cell lines, hepatocellular carcinoma cell lines J5, Huh7, and Hep3B; non-small cell lung adenocarcinoma cell line A549; stomach adenocarcinoma cell line AGS; and colon adenocarcinoma cell line WiDr were used in this study. All cell lines were treated with Ple and Ple-a, respectively, at different concentrations (0, 5, 10, 25, 50, 75, 100 μM) for 24 h, then MTT assay was performed, and 50% inhibition concentration (half maximal inhibitory concentration: IC_50_) was measured. In addition, in order to test whether these two peptides have a toxic effect on normal cells, mouse embryonic fibroblast NIH-3T3 was used as control. According to the IC_50_ values summarized in Table 4, Ple-a (IC_50_ = 11~197 μM) was significantly more potent against all cancer cell lines than Ple (IC_50_ = 54.9~>500 μM). Both peptides showed little cytotoxicity against NIH-3T3 cells (IC_50_ = 313~>500 μM). Among these cancer cell lines, non-small cell lung adenocarcinoma cell line A549 was selected for further investigation. Ple-a, which showed notable selective cytotoxicity against A549 (IC_50_ = 42 μM) and NIH-3T3 (IC_50_ = 313 μM) cell lines, was used in the rest of the study.

### 2.5. Ple-a Alters A549 Cell Morphology

**Figure 2:**
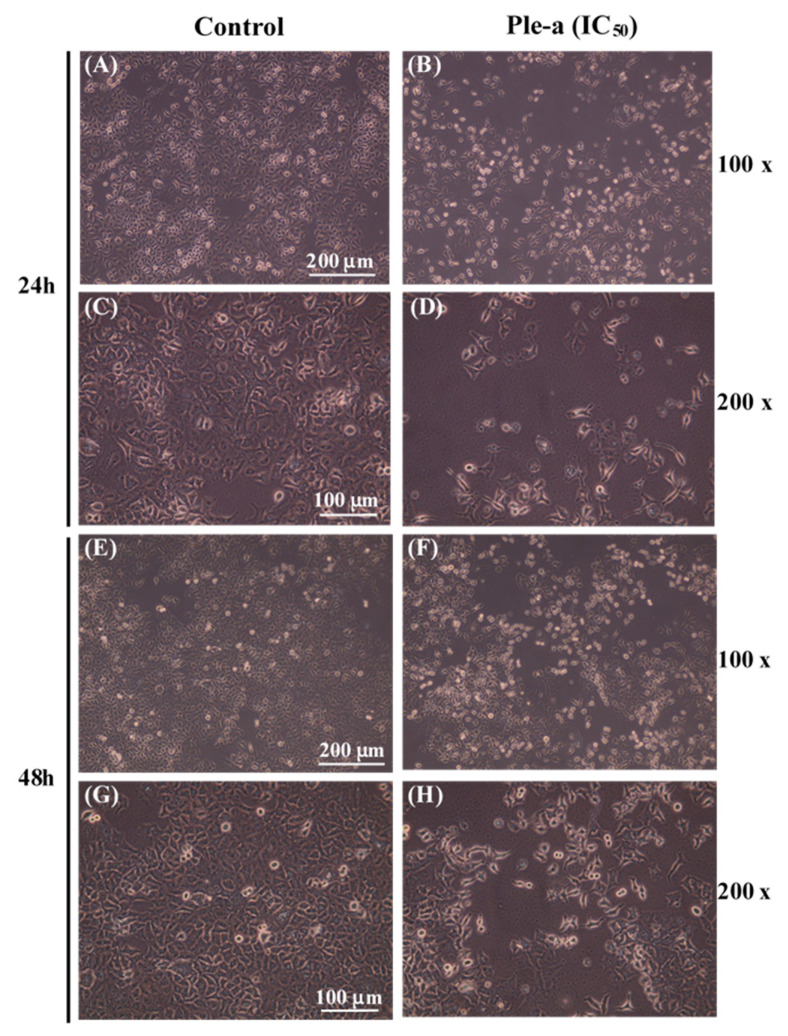
Effects of Ple-a on cell morphology changes of A549 cells. Cells were treated with Ple-a at IC_50_ of 42 μM for 24 h (**A**–**D**) and 48 h (**E**–**H**) and observed with an inverted optical microscope.

A549 cells were treated with 42 μM of Ple-a and cell morphology was assessed at 24 h and 48 h (Figure 2) using an inverted optical microscope. Upon Ple-a treatment for 24 h, it induced the appearance of membrane-bound particles, producing irregular shapes that were smaller in size than the untreated cells. A549 cells also showed obvious rounding and death, and as the magnification increased from 100× to 200×, slight cell shrinkage was observed, with vacuoles suspected of autophagosomes found in the cytoplasm; these are similar to the typical characteristics of apoptosis and autophagy, respectively. After the A549 cells had been incubated with Ple-a for 48 h, they significantly shrunk in size and lost their capacity to adhere to the substratum, while the phenomenon of vacuolation tended to decrease.

### 2.6. Ple-a Increased the Number of Cells in the Sub-G1 Phase as Revealed by Flow Cytometry

**Figure 3:**
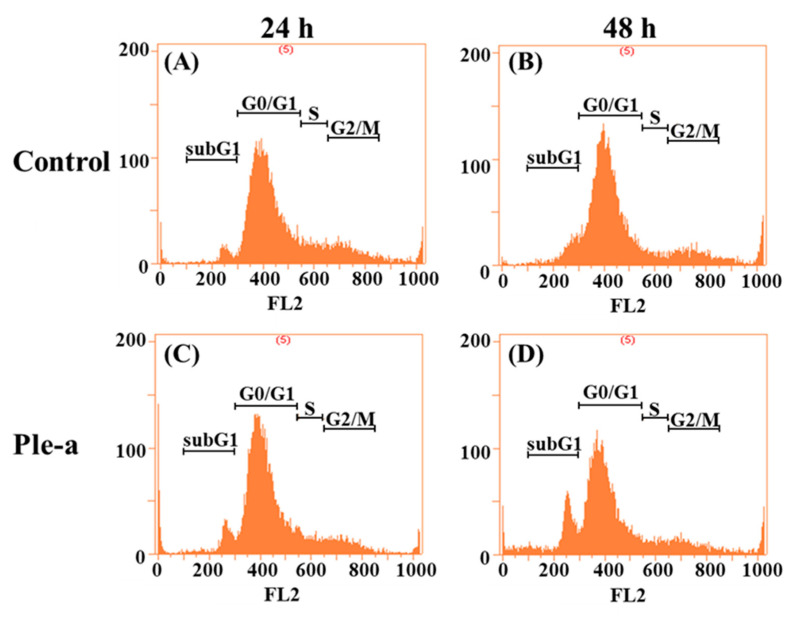
Flow cytometry was applied to monitor A549 cell cycle changes in the absence and presence of Ple-a (IC_50_) for 24–48 h, with visualization by propidium iodide (PI) staining. A549 cells treated without Ple-a for 24 h (**A**) and 48 h (**B**) and treated with Ple-a for 24 h (**C**) and 48 h (**D**).

To investigate whether Ple-a induced cytotoxicity of A549 cell lines through the apoptotic pathway, flow cytometry was applied to monitor A549 cells in the absence of presence of Ple-a for 24–48 h, with visualization by propidium iodide (PI) staining. After 24 h of Ple-a (IC_50_) treatment, it could be clearly observed that the number of cells in the sub-G1 phase increased (4.87% vs. 8.02%, Figure 3A,C), while 48 h of treatment further increased the number of cells in the sub-G1 phase to about twice that of the control group (8.97% vs. 17.05%, Figure 3B,D). An increased percentage of cells in the sub-G1 phase was observed in a time-dependent manner (Figure 3), which is indicative of apoptotic cells.

### 2.7. Ple-a Induced Both Apoptosis and Autophagy of A549 Cells at the Early Stage

**Figure 4:**
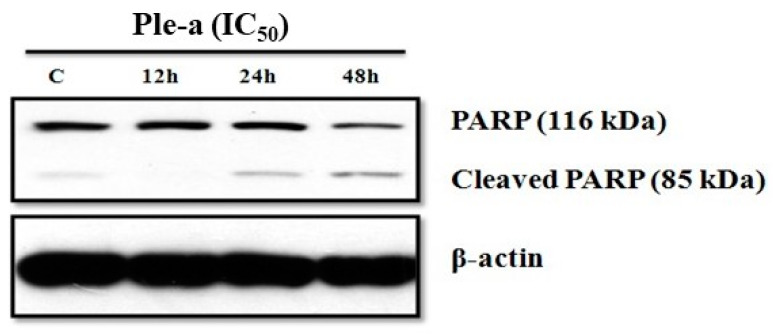
Western blot analysis was applied to monitor if poly(ADP-ribose) polymerase (PARP), the final downstream substrate of caspase-3, was cleaved in A549 cells treated with Ple-a (IC_50_) for 12–48 h.

To further investigate whether Ple-a induced apoptosis of A549 cells through the caspase-dependent pathway, we determined if poly(ADP-ribose) polymerase (PARP), the final downstream substrate of caspase-3, was cleaved in cells treated with Ple-a. According to the results of western blot analysis, the pro-form PARP (116 kDa) gradually decreased upon treatment of Ple-a for 24 to 48 h, while the cleaved form PARP (85 kDa) increased accordingly, indicating the caspase-dependent apoptotic effect (Figure 4).

**Figure 5:**
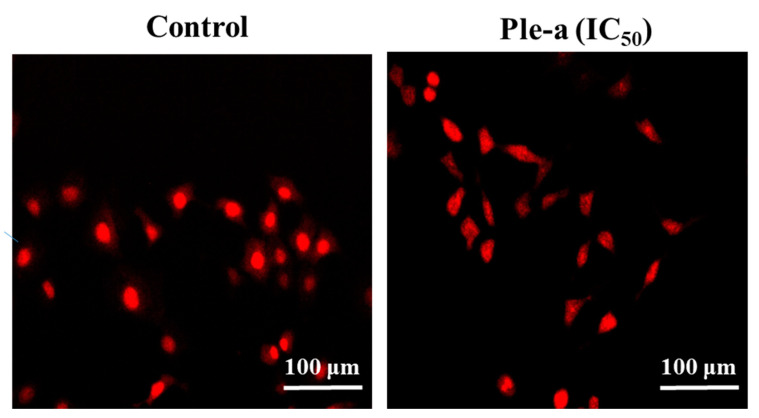
Fluorescence microscopy with PI staining; a fluorescent dye that specifically binds to DNA was applied to monitor the apoptotic effects of Ple-a treatment (42 μM, IC_50_) for 48 h on A549 cells.

Propidium iodide (PI) is a fluorescent dye that specifically binds to DNA. It is often used for DNA quantification and can also be used to observe cell morphology [35]. After A549 cells were treated with Ple-a at a concentration of 42 μM (IC_50_) for 48 h, it was found that the nucleus began to deform and appeared irregular, and chromosomes were observed to shrink and the appearance was irregular; these are also indicative of Ple-a- induced apoptotic effects (Figure 5).

**Figure 6:**
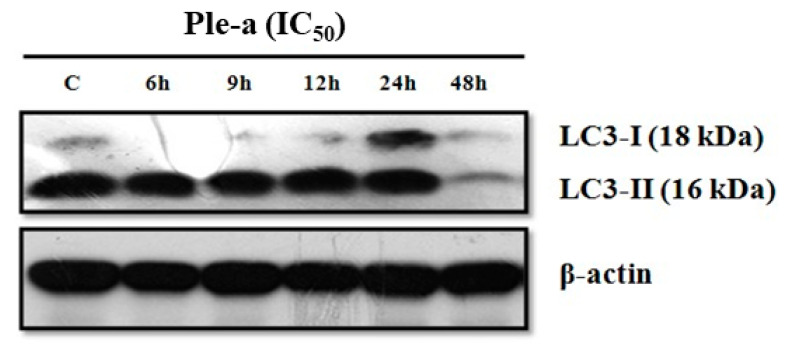
Western blot analysis was applied to monitor the expression level of autophagy-related proteins LC3-I and LC3-II in A549 cells treated with Ple-a (IC_50_) for 6, 9, 12, 24, and 48 h.

Interestingly, autophagy-related protein LC3-II was found to concomitantly increase upon Ple-a treatment for 6 to 24 h (Figure 6). These results indicated that both apoptosis and autophagy were involved in the early stage of Ple-a-induced cell death of A549 cells.

### 2.8. Ple-a-Induced Apoptosis of A549 Cells Was Enhanced by Inhibition of Autophagy at a Late Stage

**Figure 7:**
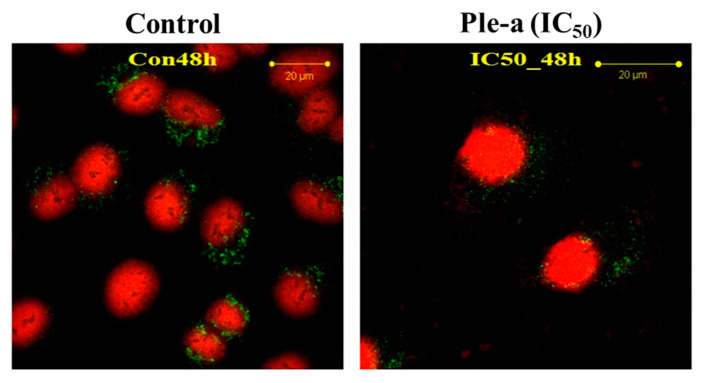
Confocal microscopy was applied to verify the in situ LC3 expression pattern. A549 cells were treated with Ple-a at a concentration of 42 μM (IC_50_) for 48 h then monitored by a confocal microscope. Red fluorescence indicated PI-stained nucleus and green fluorescence represents FITC-labelled LC3.

According to the western blot results, upon Ple-a treatment for 48 h, the expression level of LC3-II drastically decreased (Figure 6). Confocal microscopy was applied to further verify the in situ LC3 expression pattern. A549 cells were treated with Ple-a at a concentration of 42 μM (IC_50_) for 48 h and then monitored by a confocal microscope. Red fluorescence indicated PI-stained nucleus and green fluorescence represents FITC-labelled LC3. In the control group, large amounts of the green fluorescence were found to be concentrated around the nucleus, while in the Ple-a- treated group, the green fluorescence was significantly reduced and dispersed in the cytoplasm (Figure 7). Regarding the nucleus morphology, it was found that when A549 cells were not treated with Ple-a, the nucleus was intact and smooth, but after 48 h of Ple-a treatment, the nuclear membrane of the nucleus began to deform, similar to the characteristics of apoptosis (Figure 7). We therefore reasoned that Ple-a-induced apoptosis of A549 cells was enhanced by inhibition of autophagy at a late stage (48 h).

**Figure 8:**
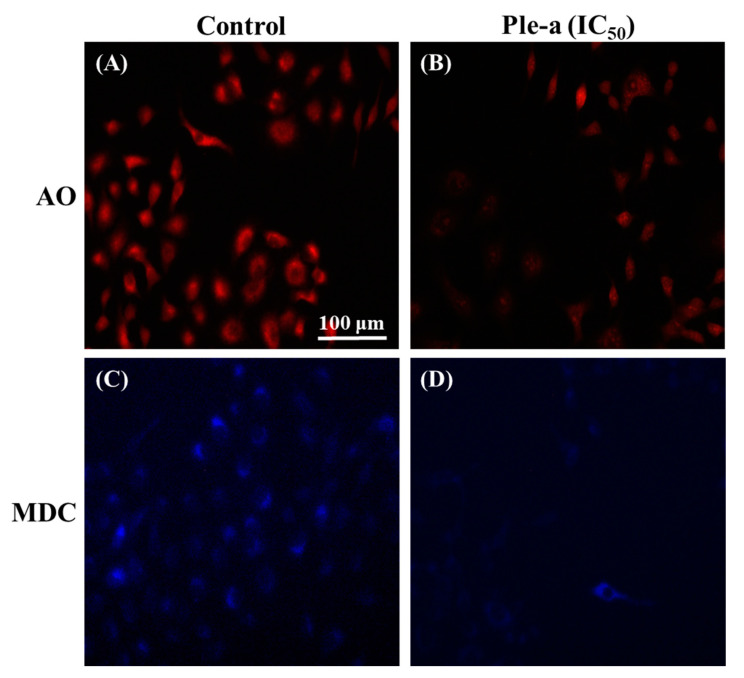
Fluorescence microscopy was applied to monitor the appearance of acidic vesicular organelles (AO staining) and the accumulation of autophagolysosomes (MDC staining) with or without Ple-a (42 μM, IC_50_) treatment for 48 h. AO staining of A549 cell treated without (**A**) or with (**B**) Ple-a; MDC staining of A549 cells treated without (**C**) or with (**D**) Ple-a.

When cells are under stress conditions, such as starvation, UV irradiation, hypoxia, and drug stimulation, autophagy is upregulated. Autophagy necessitates the formation of a double-membrane organelle termed autophagosome that ensures the capture and the transport of their contents to the acidic lysosome [36]. Acridine orange (AO) is a weak base fluorophore that accumulates in a protonated form inside acidic vesicular organelles such as lysosome [37]. In this study, A549 cells were treated with or without Ple-a at a concentration of 42 μM (IC_50_) for 48 h, and then stained with AO for 15 min, and the appearance of acidic vesicles was observed under a fluorescence microscope. It was found that after A549 cells were treated with Ple-a for 48 h, the intracellular acidic vesicles were significantly reduced (Figure 8B).

In addition to AO staining, autophagy can also be detected by another fluorescent dye, i.e., monodansylcadaverine (MDC). This fluorescent dye can specifically bind to autophagic vacuoles, thus serving as a novel in vivo autophagy marker [37]. Similarly, A549 cells were treated with Ple-a at a concentration of 42 μM (IC_50_) for 48 h and finally stained with MDC for 15 min, and the accumulation of autophagolysosomes was observed under a fluorescence microscope. According to the results in Figure 8D, the reduction of autophagosomes can be clearly found.

## 3. Discussion

Since Ple has selective lipid membrane-perturbation activities [25,26], it is a suitable candidate for use in the treatment of bacterial infections and cancer therapy. Previous works have also shown that the peptide showed cytotoxicity against breast and myeloma carcinoma cells [27,28]. However, whether Ple is effective against multidrug-resistant (MDR) bacteria remains unknown. In the current study, we intended to investigate whether Ple and its C-terminal amidated derivative Ple-a are able to exhibit antibacterial effects against MDR bacterial strains, the synergistic effects of Ple-a used in combination with conventional antibiotics, and further delineate its anticancer efficacy and mechanism against a non-small cell lung adenocarcinoma A549 cell line.

We determined the antibacterial activity of Ple and Ple-a against a broad spectrum of Gram-positive and Gram-negative bacterial strains. As shown in Table 2, Ple-a exhibited higher antibacterial activity against Gram-negative bacteria (MIC values of 0.5~16 μg/mL), as compared with those against Gram-positive strains (MIC values of 4~32 μg/mL). In a recent study using all-atom and coarse-grained molecular dynamics simulations to provide molecular-level insights into the pore-forming process [38], Ple was shown to construct a more efficient and stable pore in the anionic membranes than in the zwitterionic membranes. Ple-a may exhibit higher affinity and better antibacterial activity toward Gram-negative bacteria by perturbation and permeabilizing their outer membrane via forming either toroidal or disordered toroidal pores with different peptide arrangements.

Six classical categories for the mechanism of action of antibiotics include inhibitors of DNA replication (DNA synthesis, DNA gyrase and follic acid metabolism), RNA synthesis (DNA-directed RNA polymerase and RNA elongation), protein synthesis (50S or 30S ribosomal subunit inhibitors), cell wall biosynthesis, cell membrane biosynthesis, and fatty acid synthesis [39]. In this study, in order to investigate the combinatorial effects of Ple-a and conventional antibiotics, penicillin (Ampicillin), aminoglycosides (Amikacin and Gentamycin), carbapenems (Imipenem and Meropenem), cephalosporins (Ceftazidime and Cefotaxime), and fluoroquinolone (Levofloxacin) were selected. Among them, Ampicillin, Ceftazidime, Cefotaxime, Imipenem, and Meropenem are inhibitors of cell wall synthesis; Amikacin and Gentamicin are inhibitors of protein synthesis; and Levofloxacin is an inhibitor of DNA synthesis. It has been reported that Ple-derived AMPs inhibit RNA and protein synthesis in *E. coli* at sublethal concentrations [20]. Among all MDR bacterial strains tested, Ple-a showed the most potent antibcterial activity against *E. coli*-7R with the MIC value of 2 μg/mL (Table 2). It was found that when Ple-a and antibiotics were used at a ratio of 1:1, Ampicillin, Cefotaxime, and Gentamycin showed synergistic effects. When Ple-a and antibiotics were used at a ratio of 2:1, Ampicillin, Imipenem, Ceftazidime, Cefotaxime, and Gentamycin showed synergistic effects. However, when Ple-a and antibiotics were used at a ratio of 1:2, only Cefotaxime showed a synergistic effect (Table 3). Moreover, when Ple-a and antibiotics were used at a ratio of 1:1 or 2:1, all antibiotics except meropenem showed synergistic or additive effects. Meropenem, which is effective against *E. coli*-7R with a rather low MIC value of 0.5 μg/mL, did not show any additive or synergistic effect with Ple-a. We therefore postulated that Ple-a exhibited better antibacterial activity against the MDR strain of *E. coli* when used in combination with antibiotics of different mechanism of action.

Previous studies have demonstrated that Ple show cytotoxicity against breast and myeloma carcinoma cells [27,28]. In this study, our results revealed that Ple and Ple-a exhibited selective cytotoxicity against several typical cancer cell lines including hepatocellular carcinoma cell lines J5, Huh7, and Hep3B; non-small cell lung adenocarcinoma cell line A549; gastric adenocarcinoma cell line AGS; and colorectal adenocarcinoma cell line WiDr, and further delineate its anticancer efficacy and mechanism against the A549 cell line. First, we monitored A549 cell morphology change upon treatment with 42 μM of Ple-a for 24h and 48 h. Ple-a treatment for 24 h induced the appearance of membrane-bound particles producing irregular shapes that are smaller in size than the untreated cells; slight cell rounding and shrinkage was observed, with vacuoles suspected of autophagosomes found in the cytoplasm, and these are similar to the typical characteristics of apoptosis and autophagy, respectively (Figure 2). However, upon Ple-a treatment for 48 h, A549 cells significantly shrunk in size and lost their capacity to adhere to the substratum, while the phenomenon of vacuolation tended to decrease (Figure 2). Flow cytometry with PI staining suggested that Ple-a induced cytotoxicity of A549 cells through the apoptotic pathway, showing an increased percentage of cells in the sub-G1 phase in a time-dependent manner (Figure 3). Western blot analysis revealed that the pro-form PARP (the final downstream substrate of caspase-3, 116 kDa) gradually decreased upon treatment of Ple-a for 24 to 48 h, while the cleaved form PARP (85 kDa) increased accordingly, indicating the caspase-dependent apoptotic effect (Figure 4). PI staining further confirmed that upon Ple-a treatment for 48 h, the A549 cell nucleus began to deform and appeared irregular, and chromosomes were observed to shrink and the appearance was irregular; these are also indicative of Ple-a-induced apoptotic effects (Figure 5). Apoptotic cell death can be induced by antimicrotubule agents that are known to result in the aberrant formation of the mitotic spindle and blockage of the cell cycle in G2/M phase [40]. In a previous report [41], lactaptin, the proteolytic fragment of human milk kappa-casein, induced the death of various cultured cancer cells. Its recombinant analogue RL2 was found to directly penetrate cancer cells and its binding targets were identified as α/β-tubulin and α-actinin-1. The authors hypothesized that the interactions between RL2 and cytoskeletal proteins may result in apoptotic and autophagic cell death of cancer cells. We therefore reasoned that Ple-a may also exert its anticancer activity by binding to microtubules, destabilize complexes of focal adhesion, and induce irregular shapes or even shrinking of cancer cells. Further studies are now being conducted to delineate the intracellular binding targets of Ple-a in A549 cells.

Surprisingly, autophagy-related protein LC3-II was found to concomitantly increase upon Ple-a treatment for 6 to 24 h (Figure 6), indicating that both apoptosis and autophagy were involved in the early stage of Ple-a-induced cell death of A549 cells. However, upon Ple-a treatment for 48 h, both LC3-I and LC3-II began to decrease significantly (Figure 6). Confocal microscopy showed that in the control group, large amounts of the FITC-LC3 (green fluorescence) were found to be concentrated around the nucleus, while in the Ple-a-treated group, the green fluorescence was significantly reduced and dispersed in the cytoplasm (Figure 7). And after 48 h of Ple-a treatment, the nuclear membrane of A549 cells began to deform, indicative of apoptosis (Figure 7). AO and MDC staining further confirmed that upon Ple-a treatment for 48 h, the intracellular acidic vesicles and the accumulation of autophagolysosomes were significantly reduced (Figure 8B). We therefore reasoned that Ple-a-induced apoptosis of A549 cells was enhanced by inhibition of autophagy at a late stage (48 h). In our previous studies, a 25 a.a. peptide fragment, lactoferricin B25 (LFcinB25) exhibited potent anticancer capability against gastric adenocarcinoma AGS cell line, and exerted selective cytotoxicity against the AGS cells via enhanced caspase-dependent apoptosis by inhibition of autophagy at the final stage [42]. Additionally, a cationic AMP GW-H1 exerts highly selective cytotoxicity against AGS gastric cancer cell lines via both apoptosis and autophagy in the early stage, and the caspase-dependent apoptosis was further enhanced by inhibition of autophagy at the final stage, with beclin-1 serving as a promising target for inhibiting autophagy to sensitize GW-H1 therapy for gastric cancer [43].

## 4. Materials and Methods

### 4.1. Bacterial Strains and Culture Conditions

Seven Gram-positive and eighteen Gram-negative bacteria were selected for measurement of the antibacterial activity of AMPs used in the present study, including seven important marine pathogens kindly provided by Dr. Tsun-Yung Kuo’s lab as clinical isolates from diseased aquaculture, and several multidrug-resistant (MDR) bacterial pathogens were collected from National Yang-Ming Chiao-Tung University Hospital located in Yilan City, Taiwan, including *Enterococcus faecium*-4R, *Escherichia coli*-7R, *Pseudomonas aeruginosa*-5R, *Klebsiella pneumoniae*-7R, *Klebsiella pneumoniae*-10R, and *Acinetobacter baumannii*-8R. Another strain, *Salmonella enterica* serovar Choleraesuis-13R, was obtained from the Animal Health Research Institute, Council of Agriculture, Executive Yuan, Taiwan (as summarized in Table 2). Bacteria were cultured freshly for every experiment by cultivation from frozen stock at 37 °C for 12–14 hr in Trypton Soy Broth (TSB, Oxoid Basingstoke, UK). Glycerol stocks (20%, *v*/*v*) were maintained at −86 °C for long-term storage.

### 4.2. AMPs and Their Aantibacterial Activity

AMPs used in this study (Ple and Ple-a) were synthesized and purified by Kelowna International Scientific Inc., New Taipei City, Taiwan. The antimicrobial activities were measured by a minimal inhibitory concentration (MIC) susceptibility test according to Chou et al. [30]. Briefly, MIC was determined by incubating in 135 µL of a final inoculum of 10^4^ CFU/mL bacterial suspension with various concentrations of 15 μL AMP solution (0–256 µg/mL) tested in the 96-well microtiter plates. Cultures were examined for growth after 48 h incubation at 37 °C, and the absorbance at 600 nm was measured. The MIC value was defined as the lowest concentration of AMP that completely inhibits visible bacterial growth after 48 h incubation.

### 4.3. Checkerboard Assay for Drug Combination Effect

An aliquot of an overnight culture of *E. coli*-7R was grown in TSB diluted in 2 mL normal saline to achieve 0.5 McFarland turbidity (1.0 × 10^7^ bacterial cell CFU/mL). A volume of 500 μL of the 0.5 McFarland solution was added to 4500 mL of TSB media (this was a 1/1000 dilution). Drug combination analyses were performed using the checkerboard titration method in sterile 96-well polypropylene microtiter plates according to a previous report [34] with slight modifications. The range of antibiotics and Ple-a dilutions used were 0–256 μg/mL and were prepared by dilution with phosphate-buffered saline (pH 7.4). The clinically used antibiotics were ampicillin, amikacin, gentamycin, levofloxacin, ampicillin-sulbactam (Sigma, St. Louis, MO, USA), imipenem, meropenem (AstraZeneca, Rome, Italy), ceftazidime, and cefotaxime (Wyeth-Lederle, Aprilia, Italy). Drug solutions were prepared on the day of assay or stored in the dark at −80 °C for short time periods. Assay plates were inoculated with 100 μL of bacterial suspensions and incubated at 37 °C for 24 h. The following equation was applied to calculate the fractionary inhibitory concentration (FIC) index (FICI): A/MIC_A_ + B/MIC_B_ = FIC_A_ + FIC_B_ = FICI, where A and B are the MIC of (antibiotic + Ple-a) in combination (in a single well), MIC_A_ and MIC_B_ are the MIC of each compound individually, and FIC_A_ and FIC_B_ are the FICs of antibiotic and Ple-a, respectively. FICI was interpreted as follows: ≤0.5, synergistic; >0.5 to 1.0, additive; >1.0 to< 4.0, indifference; and ≥4.0, antagonism. All experiments were performed in triplicate.

### 4.4. Cell Lines and Culture Conditions

The human hepatocellular carcinoma cell line Hep3B (BCRC 60434) and the murine embryonic fibroblast cell line NIH-3T3 (BCRC 60071) were obtained from Bioresources Collection and Research Center (Hsin Chu, Taiwan). The human hepatocellular carcinoma cell line Huh7 (ATCC CCL-185) was obtained from American Type Culture Collection, Manassas, VA, USA. The J5 cell line of human hepatocellular carcinoma cells was kindly provided by Dr. M. J. Chou (Graduate Institute of Basic Medical Science, Chang Gung University, Tao-Yuan, Taiwan). The A549 and AGS cell lines were kindly provided by Dr. Chia-Hsien Cheng (Graduate Institute of Tumor Medicine, National Taiwan University, Taipei, Taiwan). The HepG2 cells were maintained with Dulbecco’s modified Eagle’s medium containing 10% fetal bovine serum, and the J5 cells were maintained with RPMI 1640 medium containing 10% fetal bovine serum at 37 °C in a humidified atmosphere containing 5% CO_2_, as previously described [44]. The culture conditions and protocols were according to Chen et al. [44] and Pan et al. [42,43].

### 4.5. Cell Morphology Monitoring

A549 cells were plated at a density of 3500 cells/well in 96-well plates and were permitted to adhere for 12–18 h and then washed with phosphate buffered saline (PBS). Cells were treated with 42 μM of Ple-a, and cell morphology changes were monitored at 24 and 48 h using an inverted microscope with 100× and 200× magnification.

### 4.6. Cell Viability Assay

The viability of all cell lines after treatment with Ple and Ple-a was evaluated using 3-(4,5-dimethylthiazol-2-yl)-2,5-diphenyltetrazolium bromide (MTT) assays performed in triplicate in three independent experiments according to Chen et al. [44] and Pan et al. [42,43] with slight modifications. Briefly, cells were plated at a density of 3500 cells/well in 96-well plates, and were permitted to adhere for 24 h then washed with phosphate buffered saline (PBS). Solutions were always prepared freshly by dissolving 1× PBS or Ple and Ple-a in culture medium and added to all cell lines. After 24 h of exposure, the peptide-containing medium was removed, washed with PBS, and replaced by fresh medium. The cells in each well were then incubated in culture medium with 0.5 µg/mL MTT for 2 h. After the media were removed, 150 µL of DMSO was added to each well. Absorbance at 540 nm of the maximum was detected by a multimode microplate reader SpectraMax M2 (Molecular Devices, San Jose, CA, USA). The viability of DMSO-treated cells was considered as 100%. The software Calcusyn was applied to calculate the IC_50_ values. The results were determined by three independent experiments.

### 4.7. Flow Cytometric Analysis of Cell Cycle

Briefly, A549 cells were plated at a density of 5 × 10^5^ cells/well in 6-well plates and were permitted to adhere for 24 h. After treatment with Ple-a of 42 μM (IC_50_) for 24 and 48 h, cells were harvested and washed twice with PBS and fixed in 70% cold ethanol at 4 °C overnight. Before analysis, cells were washed twice with PBS containing 1% BSA, resuspended with 400 μL of PBS, and treated with 100 μg/mL RNase A (Roche Diagnostics, Indianapolis, IN, USA) and 20 μg/mL propidium iodide (PI; Sigma Corp., St. Louis, MO, USA). After incubation for 30 min at 37 °C, the cells were subjected to DNA content analysis. The PI fluorescence was analyzed by using a FACScalibur flow cytometer (Becton Dickinson, Franklin Lakes, NJ, USA). Data from at least 10,000 cells were analyzed with FlowJo software v7.6.5 (Becton Dickinson, Ashland, OR, USA). Cell cycle distributions were calculated with FlowJo software.

### 4.8. Western Blot Analysis

Approximately 7 × 10^5^ cells were cultured in 60 mm^2^ dishes and then incubated with 42 μM Ple-a for the indicated time. The cells were lysed on ice with 200 μL of protein extraction buffer (50 mM Tris-HCl, pH 7.5, 0.5 M NaCl, 5 mM MgCl_2_, 0.5% Nonidet P-40, 1 mM phenylmethylsulfonyl fluoride, 1 μg/mL pepstatin, and 50 μg/mL leupeptin) and centrifuged at 12,000× g at 4 °C for 10 min. The protein concentration of the cell lysates was measured with a Bio-Rad protein assay (Bio-Rad Laboratories, Hercules, CA, USA) following the manufacturer’s instructions. Aliquots (20 μg) of the cell lysates were separated by 12.5–15% sodium dodecyl sulfate-polyacrylamide gel electrophoresis (SDS-PAGE; Bio-Rad, Hercules, CA, USA). Resolved proteins were then transferred to polyvinyldenefluoride (PVDF) membranes. Filters were blocked with 5% non-fat milk overnight and 1:500~1:1000 dilutions of primary antibodies (mouse anti-PARP IgG, and mouse anti-β-actin IgG, Santa Cruz Biotechnology, Dallas, TX, USA) for 1 h at room temperature. Membranes were washed with three times with 0.05% Tween-20 and incubated with a 1:5000 dilution of HRP-conjugated secondary antibody (goat-anti-mouse IgG-HRP, Santa Cruz Biotechnology, Dallas, TX, USA) for 1 h at room temperature, and then visualized with an enhanced chemiluminescence (ECL) plus chemiluminescence system (Millipore, Billerica, MA, USA).

### 4.9. Fluorescent Staining and Confocal Microscopy

To monitor Ple-a induced autophagic and apoptotic cell death events, 5 × 10^4^ A549 cells were seeded on glass cover slips in 6-well plates and treated with 42 μM Ple-a for 48 h. Routinely, cells were washed with PBS, fixed with ice cold 4% paraformaldehyde for 30 min at 4 °C and blocked with 5% BSA in PBS for 1 h. Cells were incubated with primary antibody to LC3 (1:1000 dilution anti-Rabit-LC3-pAb, Novus Biologicals, Centennial, CO, USA) overnight at 4 °C and then incubated with FITC-goat-anti-rabbit-IgG (Santa Cruz Biotechnology, Dallas, TX, USA) for 30 min at 37 °C in the dark; PI (Sigma Corp., St. Louis, MO, USA) was used for nuclear staining. After extensive washing, the cover slips were then mounted on glass slides and the fluorescent images were captured with confocal microscopy (Olympus FV1000 laser confocal microscope, Tokyo, Japan). Furthermore, A549 cells were treated with or without Ple-a at a concentration of 42 μM (IC_50_) for 48 h, stained with acridine orange (AO) or another fluorescent dye monodansylcadaverine (MDC) for 15 min [37], and the appearance of acidic vesicles and the accumulation of autophagolysosomes was observed under a fluorescence microscope (Zeiss Axio Obserrer D1, Jena, Germany).

### 4.10. Statistical Analysis

Data were expressed as mean values plus standard deviations. One-way ANOVA combined Bonferroni’s multiple comparison test using SAS software (version 9.4, 2012; SAS Institute, Cary, NC, USA) was used to specify the differences between groups. A *p* value of <0.05 was considered statistically significant. *p* value: *, <0.05; **, <0.01; ***, <0.001.

## 5. Conclusions

In this study, we used a potent AMP called Pleurocidin (Ple) derived from winter flounder *Pleuronectes americanus* and its C-terminal-amidated derivative Pleurocidin-amide (Ple-a) and evaluated their antibacterial and anticancer activities. Our results indicated that both Ple and Ple-a exhibited significant antibacterial activity against a broad spectrum of Gram-positive and Gram-negative bacteria, especially marine pathogens. These peptides are also potent against several clinical isolates of multidrug-resistant (MDR) bacterial strains. When used in combination with several conventional antibiotics with different mechanisms of action, they exhibited synergistic and additive effects against MDR *E. coli*. Ple and Ple-a also showed notable cytotoxicity toward various cancer cell lines, while normal mouse fibroblast 3T3 cells were less susceptible to these peptides. Ple-a exhibited selective cytotoxicity against A549 human lung adenocarcinoma cells via both apoptosis and autophagy in the early stage, and the caspase-dependent apoptosis was further enhanced by inhibition of autophagy at the final stage. Our findings provided support for future applications of Ple-a as a potential therapeutic agent for bacterial infections and cancer treatment.

## Figures and Tables

**Table 1:** Sequence characteristics of Pleurocidin (Ple) and Pleurocidin-amide (Ple-a).

AMP	Amino Acid Sequence	Charge	MW	Hyrophobicity ^a^	Hydrophobic Moment ^a^
Ple	GWGSFF**KK**AA**H**VG**KH**VG**K**AALT**H**YL	+7	2711.17	−0.026	0.287
Ple-a	GWGSFF**KK**AA**H**VG**KH**VG**K**AALT**H**YL-NH_2_	+8	2710.18	−0.026	0.287

^a^ The hydrophobicity values used in this study were based on the “Consensus scale”, and the hydrophobic moment was calculated accordingly [33].

**Table 2:** Antimicrobial activity of Ple and Ple-a against typical Gram-positive and Gram-negative bacterial strains, including clinically isolated marine bacterial pathogens.

Bacterial Strain		G(+)/G(−)	MIC (μg/mL) ^a^	
			Ple	Ple-a
**Typical G(+) and** **G(−) bacterial** **strains**	*Staphylococcus aureus*	G(+)	16	4
	*Staphylococcus xylosus*	G(+)	16	8
	*Listeria monocytogenes*	G(+)	32	16
	*Streptococcu bovis*	G(+)	128	32
	*Escherichia coli*	G(−)	16	2
	*Enterobacter aerogenes*	G(−)	2	1
	*Enterobacter cloacae*	G(−)	4	1
	*Yersinia enterocolitica*	G(−)	32	8
	*Pseudomonas aeruginosa*	G(−)	8	2
	*Salmonella enterica*	G(−)	64	8
	*Klebsiella oxytoca*	G(−)	8	2
**Multidrug-resistant** **G(+) and G(−)** **bacterial strains**	*Enterococcus faecium*-4R	G(+)	256	32
	*Escherichia coli*-7R	G(−)	16	2
	*Pseudomonas aeruginosa*-5R	G(−)	64	16
	*Klebsiella pneumoniae*-7R	G(−)	64	8
	*Klebsiella pneumoniae*-10R	G(−)	128	8
	*Salmonella enterica* serovar Choleraesuis-13R	G(−)	32	8
	*Acinetobacter baumannii*-8R	G(−)	8	4
**Clinically isolated** **marine bacterial** **pathogens**	*Streptococcus iniae*	G(+)	128	32
	*Lactococcus garvieae*	G(+)	128	32
	*Vibrio alginolyticus*	G(−)	8	1
	*Vibrio harveyi*	G(−)	8	2
	*Vibrio parahaemolyticus*	G(−)	8	1
	*Vibrio anguillarum*	G(−)	128	32
	*Photobacterium damselae subsp. piscicida*	G(−)	1	0.5

^a^ Antimicrobial activity (minimal inhibitory concentration, MIC) is given as the geometric mean of three sets of determinations.

**Table 3:** Combination effect of Ple-a with conventional antibiotics against MDR *E. coli*.

		Ple-a:Antibiotic (1:1)		
	MICo	MICc (1:1)	FICI	Type of Interaction
Ple-a	2			
Ampicillin	256			
Ple-a/Ampicillin		1/1	0.50	Synergistic
Imipenem	16			
Ple-a/Imipenem		1/1	0.56	Additive
Meropenem	0.5			
Ple-a/Meropenem		0.5/0.5	1.25	Indifference
Ceftazidime	128			
Ple-a/Ceftazidime		1/1	0.51	Additive
Cefotaxime	256			
Ple-a/Cefotaxime		1/1	0.50	Synergistic
Amikacin	16			
Ple-a/Amikacin		1/1	0.56	Additive
Gentamycin	256			
Ple-a/Gentamycin		1/1	0.50	Synergistic
Levofloxacin	2			
Ple-a/Levofloxacin		1/1	1.0	Additive
Ampicillin-sulbactam	128			
Ple-a/Ampicillin-sulbactam		1/1	0.51	Additive
		**Ple-a:Antibiotic (2:1)**		
	**MICo**	**MICc (2:1)**	**FICI**	**Type of interaction**
Ple-a	2			
Ampicillin	256			
Ple-a/Ampicillin		0.5/0.5	0.25	Synergistic
Imipenem	16			
Ple-a/Imipenem		0.5/0.5	0.28	Synergistic
Meropenem	0.5			
Ple-a/Meropenem		0.5/0.5	1.25	Indifference
Ceftazidime	128			
Ple-a/Ceftazidime		0.5/0.5	0.25	Synergistic
Cefotaxime	256			
Ple-a/Cefotaxime		0.5/0.5	0.25	Synergistic
Amikacin	16			
Ple-a/Amikacin		1/1	0.56	Additive
Gentamycin	256			
Ple-a/Gentamycin		1/1	0.50	Synergistic
Levofloxacin	2			
Ple-a/Levofloxacin		1/1	1.0	Additive
Ampicillin-sulbactam	128			
Ple-a/Ampicillin-sulbactam		1/1	0.51	Additive
		**Ple-a:Antibiotic (1:2)**		
	**MICo**	**MICc (1:2)**	**FICI**	**Type of interaction**
Ple-a	2			
Ampicillin	256			
Ple-a/Ampicillin		2/2	1.01	Indifference
Imipenem	16			
Ple-a/Imipenem		1/1	0.56	Additive
Meropenem	0.5			
Ple-a/Meropenem		0.5/0.5	1.25	Indifference
Ceftazidime	128			
Ple-a/Ceftazidime		2/2	1.02	Indifference
Cefotaxime	256			
Ple-a/Cefotaxime		1/1	0.50	Synergistic
Amikacin	16			
Ple-a/Amikacin		2/2	1.13	Indifference
Gentamycin	256			
Ple-a/Gentamycin		2/2	1.01	Indifference
Levofloxacin	2			
Ple-a/Levofloxacin		1/1	1.0	Additive
Ampicillin-sulbactam	128			
Ple-a/Ampicillin-sulbactam		2/2	1.02	Indifference

MICo, MIC of one sample alone; MICc, MIC of one sample in the combination. FICI (Fractional inhibition concentration index) = (MIC of Ple-a in the combination/MIC of Ple-a only) + (MIC of antibiotic in the combination/MIC of antibiotic) [34]. The FIC indexes were interpreted as follows: ≤0.5: Synergistic; 0.5~1: Additive; 1~4: Indifference; ≥4: Antagonism.

**Table 4:** Effects of Ple and Ple-a on cell viability in various cancer cell lines and mouse fibroblast cell line NIH-3T3.

Cell line	Description	IC_50_ (μM) ^a^	
		Ple	Ple-a
J5	Hepatocellular carcinoma	54.9	11.0
Huh7	Hepatocellular carcinoma	n.d. ^b^	60.0
Hep3B	Hepatocellular carcinoma	340.9	77.5
A549	Non-small cell lung adenocarcinoma	300.8	42.1
AGS	Stomach adenocarcinoma	186.5	29.8
WiDr	Colon adenocarcinoma	n.d.	197.3
NIH-3T3	Mouse fibroblast	n.d.	313

^a^ IC_50_ values: half maximal inhibitory concentration monitored by MTT assay and assessed by logarithmic extrapolation. ^b^ n.d.: not determined or IC_50_ values > 500 μM.

## Data Availability

Not applicable.
